# Post cardiac injury syndrome successfully treated with medications: a report of two cases

**DOI:** 10.1186/s12872-021-02200-5

**Published:** 2021-08-14

**Authors:** Mu-Shiang Huang, Yan-Hua Su, Ju-Yi Chen

**Affiliations:** 1 Division of Cardiology, Department of Internal Medicine, National Cheng Kung University Hospital, College of Medicine, National Cheng Kung University, 138 Sheng-Li Road, Tainan, 704 Taiwan; 2 Post Graduate Residency Program, Education Center, Department of Neurosurgery, National Cheng Kung University Hospital, Taipei Veteran General Hospital, Taipei City, Taiwan

**Keywords:** Pericarditis, Injury syndrome, Pacemaker, Coronary intervention

## Abstract

**Background:**

Post cardiac injury syndrome (PCIS) is induced by myocardial infarction or cardiac surgery, as well as minor insults to the heart such as percutaneous coronary intervention (PCI), or insertion of a pacing lead. PCIS is characterized by pericarditis after injury to the heart. The relatively low incidence makes differential diagnosis of PCIS after PCI or implantation of a pacemaker a challenge. This report describes two typical cases of PCIS.

**Case presentation:**

The first patient presented with signs of progressive cardiac tamponade that occurred two weeks after implantation of a permanent pacemaker. Echocardiography confirmed the presence of a moderate amount of newly-formed pericardial effusion. The second patient underwent PCI for the right coronary artery. However, despite an uneventful procedure, the patient experienced dyspnea, tightness of chest and cold sweats, and bradycardia two hours after the procedure. Echocardiography findings, which showed a moderate amount of newly-formed pericardial effusion, suggested acute cardiac tamponade, and compromised hemodynamics. Both patients recovered with medication.

**Conclusion:**

These cases illustrated that PCIS can occur after minor myocardial injury, and that the possibility of PCIS should be considered if there is a history of possible cardiac insult.

## Background

Dressler syndrome, also known as post cardiac injury syndrome (PCIS), is characterized by the development of pericarditis with or without pleural effusion, days to weeks after a myocardial infarction. In addition to myocardial infarction, PCIS has been shown to be induced by pericardiotomy and blunt trauma, as well as by minor insults to the heart, such as coronary intervention, insertion of pacemaker leads, or radiofrequency ablation [1–5]. However, since PCIS induced by insertion of a pacemaker or by coronary intervention is relatively uncommon, it is possible to miss this as an important differential diagnosis. Here, we describe two typical cases of PCIS.

## Case presentation

### Illustrative Case #1

A 78-year-old man diagnosed with sick sinus syndrome received a permanent pacemaker (DDDR-MRI Medtronic [ventricular lead (MEDTRONIC cistofix 5076–58 cm); atrial lead (MEDTRONIC cistofix 5076–52 cm)]). The procedure itself and the post-procedure screening were both uneventful. The wound was clean, and there was no evidence of pneumothorax or hemothorax on the chest X radiography (CXR). The patient was discharged on the second day after the procedure.

However, the patient complained of a productive cough during a routine outpatient clinic follow-up visit conducted a week after the procedure. Laboratory findings showed a decrease in hemoglobin level from 10.9 to 8.9 mg/dL. A CXR revealed newly developed borderline cardiomegaly and bilateral blunting of the diaphragmatic angle. Pacemaker interrogation results showed normal threshold and impedance of both leads. An evaluation performed at fourteen days after the procedure showed evidence of orthopnea, dyspnea upon exertion, and a minor fever (38 °C). Physical examination showed engorgement of the jugular vein, basal crackles over the bilateral lower lung fields, and pitting edema in both legs. Laboratory data revealed worsening anemia (decrease in hemoglobin level from 8.9 to 7.7 mg/dL), high NT-proBNP (1610 pg/ml), and hyponatremia (Na: 115 meq/L, Osmo: 267), but no leukocytosis or worsening of renal function. An echocardiography revealed moderate pericardial effusion (maximal thickness 1.5 cm) without dynamic diastolic collapse of the right ventricle (RV).

**Fig. 1:**
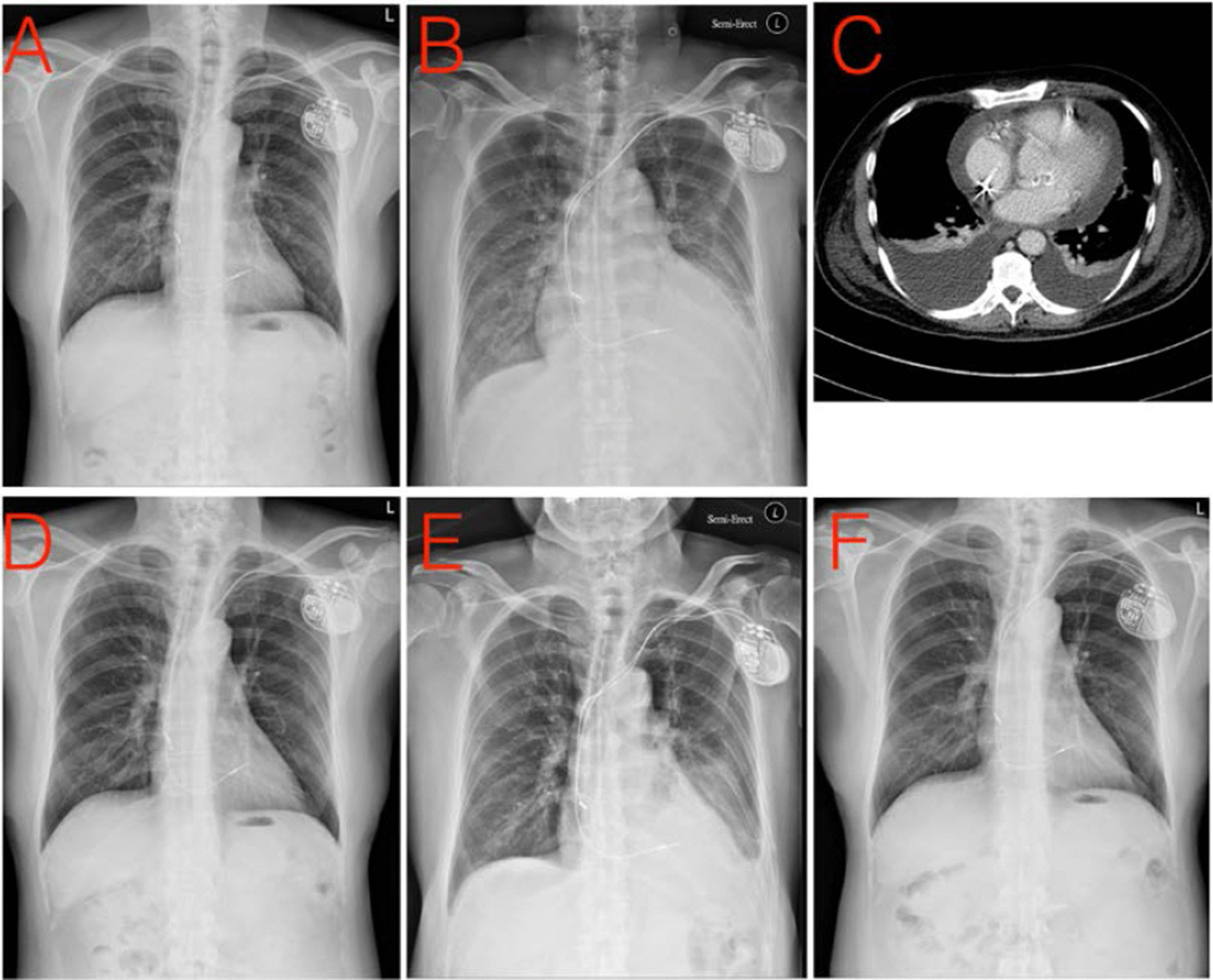
Case 1 series images. **a** One day after implantation of pacemaker; **b** significant increase in cardiothoracic ratio at fourteen days after implantation; **c** CT scan showed no lead perforation, no pericardial effusion or pleural effusion; **d** After steroid treatment; **e** Recurrent PCIS, two weeks after completion of the steroid course; **f** Fourteen days after treatment with steroids and colchicine

The patient was treated for congestion, and administered a nitrate infusion. The patient also received empirical treatment with antibiotics since the low-grade fever was suggestive of an infection. The workup for pleural effusion showed evidence of transudate, and culture results for microorganisms tested were negative, including common aerobic and anaerobic bacterium as well as mycobacterium tuberculosis. Based on concerns about lead migration or protrusion-induced hemopericardium, the patient was subjected to chest computed tomography (CT), and no protrusion was detected (Fig. 1). Additionally, the density of pericardial effusion was 20 Hounsfield unit (HU), which also did not support the possibility of hemopericardium. There was a limited improvement in the patient’s condition following several days of treatment, and echocardiography showed no worsening of pericardial effusion (thickness around 1.1 cm). Based on a suspicion of PCIS, the patient was prescribed prednisolone (30 mg/day initially), after which there was a dramatic improvement of his condition. The dosage of prednisolone was gradually tapered off to 20 mg/day after a week, 20 mg to 10 mg/day after two weeks, and the patient was maintained on 10 mg/day for 8 weeks. Although the CXR returned to baseline after completion of the steroid treatment (Fig. 1), there was a recurrence of pleural effusion, leading to the resumption of low dose prednisolone treatment in addition to colchicine treatment. The patient recovered well after this treatment.

### Illustrative Case #2

**Fig. 2:**
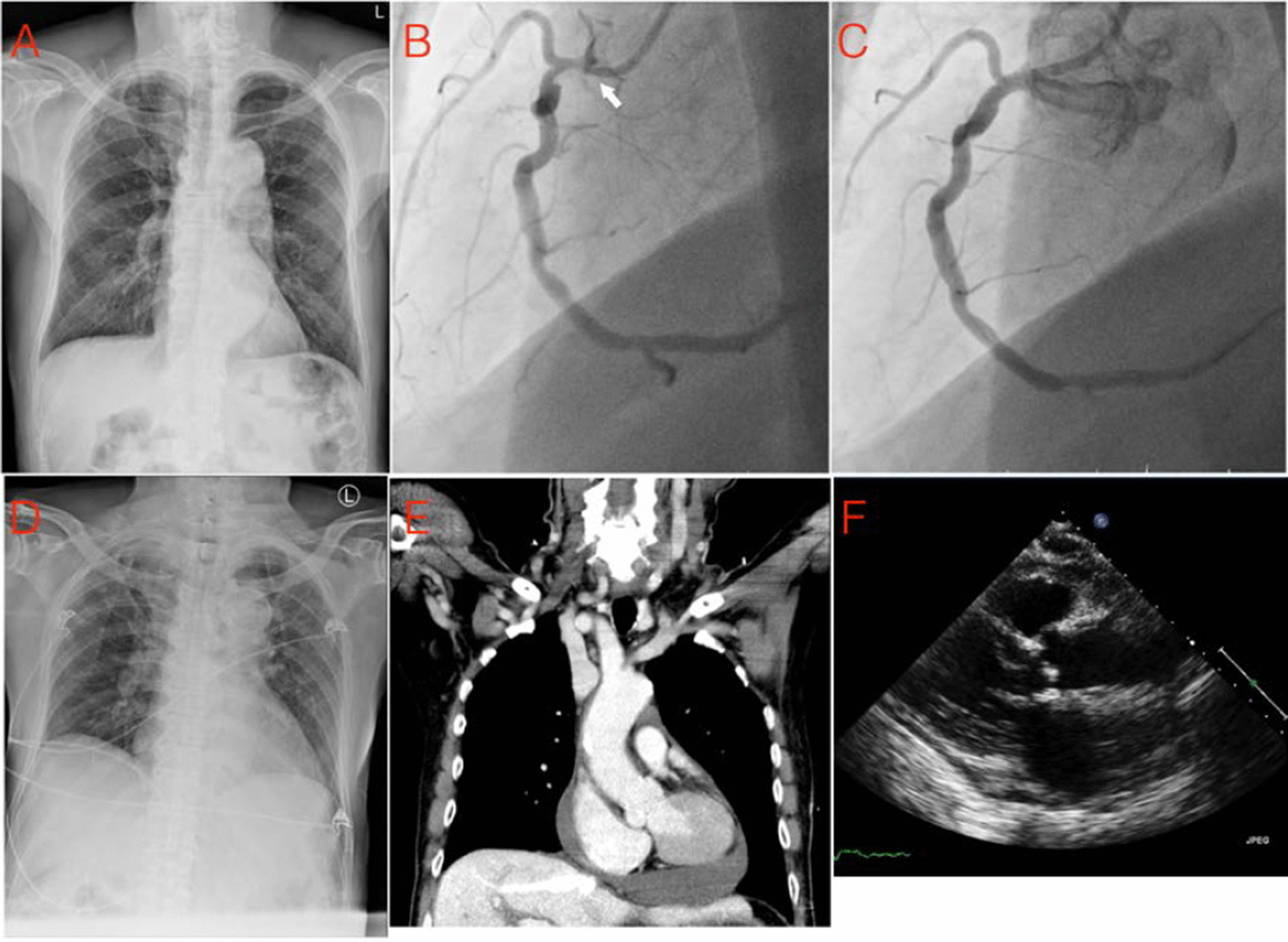
Case 2 series images. **a** Normal chest radiography (CXR) before intervention; **b** White arrow: narrow portion of right coronary artery orifice; **c** Stent deployed at orifice, no extravasation found; **d** CXR at 2 h after stent deployment; **e** chest computed tomography (CT) showed no dissection, but a significant amount of pericardial effusion; **f** Follow-up echocardiography after steroid treatment showed decreased pericardial effusion without signs of RV collapse

An 82-year-old male with a history of chronic atrial fibrillation (CHADS_2_: 3) was treated with rivaroxaban. The patient presented at the cardiology clinic with crescendo angina, and was reviewed with a coronary angiography examination, which showed significant focal stenosis at the orifice of the right coronary artery (RCA) (Fig. 2). The patient was subjected to percutaneous coronary intervention (PCI), where a Xience Xpedition stent (3.5/15 mm) was deployed at the orifice. The procedure was uneventful, and the final angiogram showed good RCA flow, and no evidence of perforation, dissection, or extravasations (Fig. 2).

However, two hours after the procedure, the patient developed cold sweats, tightness in the chest, and hemodynamic compromise along with bradycardia (50 bpm) and hypotension (systolic blood pressure: 70–80 mm Hg). Although a complete electrocardiography (ECG) showed no abnormalities, echocardiography showed new pericardial effusion (thickness 1.1–1.5 cm) with RV early diastolic collapse. This was immediately followed by a repeat coronary angiography, which showed good RCA flow without any extravasation, perforation or thrombosis. A chest CT scan with contrast showed no aortic dissection, and the density of pericardial effusion suggested hemopericardium (HU: 40) (Fig. 2). In addition to vasopressors which were administered to stabilize the hemodynamics, the patient was also prescribed a 50 mg dose of hydrocortisone for PCIS. There was an improvement in the patient’s condition on the second day and all vasopressors was discontinued in 18 h. The patient was discharged to the outpatient department with a prescription for 20 mg prednisolone, and remained stable at his follow-up visits. However, 9 months later, after we tapered off prednisolone, the patient had increased discomfort of dyspnea on exertion and the echocardiography showed pericardial effusion recurred. We prescribed colchicine and low dose prednisolone for its control and patient’s discomfort improved.

## Discussion and conclusions

PCIS is also known as pericarditis after pericardium injury. The incidence of PCIS in adults and children has been reported to be 10–40% after cardiac surgery [1, 2], about 1–5% after implantation of intra-cardiac devices [1, 3, 4], and around 0.2% after coronary intervention [5]. Our cases were consistent with previous reports which showed that the onset of PCIS could vary from hours or days, and could occur up to 5–56 days after the procedure [6, 7]. It is therefore crucial to recognize and diagnose PCIS while taking the patient’s medical history, and the patient should be evaluated at regular follow-up visits for 1–3 months after the procedure [8].

Although the clinical manifestations of PCIS are non-specific, there are several common indicators that should be closely monitored. Most patients present with fever (92%), dyspnea (78%), chest pain (50%), elevation of C-reactive protein (CRP) or erythrocyte sedimentation rate (ESR) (80%) and pleural effusion (61%) [8]. Other findings include hyponatremia, unexplained anemia, or atrial fibrillation. Although pericardial effusion is observed in almost all PCIS patients, not all patients with pericardial effusion are symptomatic or require treatment. A recent study of 968 patients showed that when echocardiography was performed 24 h after implantation of a permanent pacemaker, 98 patients had some degree of pericardial effusion, while only 19 of the patients were symptomatic and 14 required an intervention. The remaining 79 patients remain asymptomatic and did not require treatment, and most of them were free of pericardial effusion after three months [9].

In our first patient, the cause of heart failure, pericardial effusion and pleural effusion was initially unknown, and device interrogation showed normal results. The patient had low-grade fever without leukocytosis. Blood culture and thoracocentesis were performed in order to rule out infection and the possibility of hemothorax. Although there was no progression of pericardial effusion or signs of tamponade on echocardiography, chest CT scan was performed to make sure there was no perforation of the pacing leads, or venous thrombosis by the leads. This procedure was similar to a previous study by Farbod et al*.* [8], where a diagnostic flow chart was designed to rule out infection, perforation, pulmonary embolism, and lead perforation before diagnosing PCIS caused by implantation of a cardiac device.

Our second patient showed acute pericardial effusion with unstable vital signs. However, there was no direct clinical evidence which could indicate the cause of pericardial effusion. Coronary angiography and chest CT scan were repeated immediately in order to exclude all possible causes of pericardial effusion, including coronary perforation, aortic dissection, or ventricle perforation. It has been reported that patients with stable hemodynamics who have pericardial effusion can be managed conservatively and tried in patients in whom ECG, cardiac enzyme levels, and inflammatory markers indicate typical pericarditis [10, 11]. The following course of pericardial effusion recurrence 9 months later also supported PCIS etiology, rather than micro-perforation alone.

In our patients, hypervolemic hyponatremia was treated by diuresis, water restriction and increased salt intake. The three possible mechanisms which are thought to cause hyponatremia in PCIS include (1) increased secretion of ADH [12, 13], (2) elevation of atrial natriuretic factor [12], and (3) decreased effective volumes due to pericardial effusion which further decrease the glomerulus filtration rate [14]. Despite these different mechanisms, sodium levels can be corrected by simply treating the PCIS, and relieving the pericardial effusion has been shown to be sufficient to improve sodium levels without any other therapy [12, 14].

A number of studies have investigated risk factors for PCIS after pacemaker implantation. A study of 4280 patients who underwent pacemaker implantation reported that patients with a temporary transvenous pacemaker or steroid use within 7 days prior to implantation were more likely to have PCIS [15]. In addition, there was a higher risk of PCIS with active fixation in the right atrium (RA) (lateral, anterolateral side, or appendage) [9, 16]. A study of 1021 pacemaker implantations showed that there were no reports of PCIS after passive fixation or active fixation in the ventricle [17]. Female gender and antiplatelet therapy were also considered as risk factors for PCIS after pacemaker implantation [9]. In contrast, the risk factors of PCI-induced PCIS remain poorly understood, probably due to the low incidence rate.

The pathophysiology of PCIS remains unclear, and several studies suggested that it is an autoimmune reaction related to anti-myocardial antibodies resulting from injury to the myocardium or pericardium [1]. The autoimmune nature of PCIS is supported by clinical features such as the latent period between the insult and symptoms, elevation of inflammatory markers, good response to NSAIDs, and a tendency to recur. However, unlike other autoimmune diseases, circulating anti-heart antibodies are detected within 14 days of PCIS rather than at the time of diagnosis [18], and therefore do not help in PCIS diagnosis.

Both of our patients were treated with corticosteroids instead of NSAIDs, since NSAIDS were contraindicated in both patients. However, aspirin, NSAID, steroid, and colchicine have been reported as the main treatment options in a number of current case reports describing pacemaker- or PCI-induced PCIS [8, 19], and the dosage and duration of treatment are presented in Table 1 [20, 21]. Corticosteroids are usually considered as second-line treatment for patients who show a poor response to NSAIDS, or when NSAID use is contraindicated due to higher adverse event rates, longer disease duration, and higher recurrence rate [6, 20, 21]. When patients are symptom-free and exhibit normalized levels of inflammatory markers, a slow tapering-down of the steroid is recommended rather than an accelerated decrease [21]. In our case 1, the patient was administered colchicine after he had recurrent PCIS. Some case reports described simultaneous administration of colchicine along with NSAIDs or corticosteroid from the beginning, since colchicine has proved to be useful in treating refractory or recurrent post- pericardiotomy syndrome, and in lowering the recurrence rate [22]. However, if the patient does not respond to second-line treatment with steroid plus colchicine, then a combination of NSAIDs, steroids and colchicine should be considered as the next treatment option [21]. There is also one recent reported PCIS case rapidly occurred after pacemaker implantation, which was solved by prednisolone and colchicine successfully [23].

**Table 1:** Suggested medication and dosage for post cardiac injury syndrome

Medication	Dosage	Duration
Ibuprofen	600 mg tid	1–2 weeks for acute case, and 2–4 weeks for recurrent cases
Indomethacin	25–50 mg tid	
Aspirin	750–1000 mg tid	
Corticosteroid	0.2–0.5 mg/kg per day	2–4 weeks of treatment before slow tapering off
Colchicine	BW > 70 kg: 0.5 mg bid BW < 70 kg: 0.5 mg qd	3 months for acute PCIS, 6 months for recurrent PCIS

*BW* body weight

PCIS can have a good prognosis, and patients with PCIS can be treated conservatively using NSAIDs, steroids, and colchicine. Diagnosis of PCIS is usually based on exclusion of other possible causes of pericarditis.

## Data Availability

Not applicable.

## Abbreviations

CXR: Chest X radiography
CT: Computed tomography
ECG: Electrocardiography
HU: Hounsfield unit
PCI: Percutaneous coronary intervention
PCIS: Post cardiac injury syndrome
RCA: Right coronary artery
RV: Right ventricle
